# Common Genetic Variants Found in HLA and KIR Immune Genes in Autism Spectrum Disorder

**DOI:** 10.3389/fnins.2016.00463

**Published:** 2016-10-20

**Authors:** Anthony R. Torres, Thayne L. Sweeten, Randall C. Johnson, Dennis Odell, Jonna B. Westover, Patricia Bray-Ward, David C. Ward, Christopher J. Davies, Aaron J. Thomas, Lisa A. Croen, Michael Benson

**Affiliations:** ^1^Center for Persons with Disabilities, Utah State UniversityLogan, UT, USA; ^2^Biology Department, Utah State UniversityBrigham City, UT, USA; ^3^BSP CCR Genetics Core, SAIC-Frederick, Inc., Frederick National Laboratory for Cancer ResearchFrederick, MD, USA; ^4^Center for Integrated BioSystems, Utah State UniversityLogan, UT, USA; ^5^Division of Research, Kaiser Permanente of Northern CaliforniaOakland, CA, USA

**Keywords:** autism, HLA, KIR, haplotype, common genetic variant

## Abstract

The “common variant—common disease” hypothesis was proposed to explain diseases with strong inheritance. This model suggests that a genetic disease is the result of the combination of several common genetic variants. Common genetic variants are described as a 5% frequency differential between diseased vs. matched control populations. This theory was recently supported by an epidemiology paper stating that about 50% of genetic risk for autism resides in common variants. However, rare variants, rather than common variants, have been found in numerous genome wide genetic studies and many have concluded that the “common variant—common disease” hypothesis is incorrect. One interpretation is that rare variants are major contributors to genetic diseases and autism involves the interaction of many rare variants, especially in the brain. It is obvious there is much yet to be learned about autism genetics. Evidence has been mounting over the years indicating immune involvement in autism, particularly the HLA genes on chromosome 6 and KIR genes on chromosome 19. These two large multigene complexes have important immune functions and have been shown to interact to eliminate unwanted virally infected and malignant cells. HLA proteins have important functions in antigen presentation in adaptive immunity and specific epitopes on HLA class I proteins act as cognate ligands for KIR receptors in innate immunity. Data suggests that HLA alleles and KIR activating genes/haplotypes are common variants in different autism populations. For example, class I allele (HLA-A2 and HLA-G 14 bp-indel) frequencies are significantly increased by more than 5% over control populations (**Table 2**). The HLA-DR4 Class II and shared epitope frequencies are significantly above the control populations (**Table 2**). Three activating KIR genes: 3DS1, 2DS1, and 2DS2 have increased frequencies of 15, 22, and 14% in autism populations, respectively. There is a 6% increase in total activating KIR genes in autism over control subjects. And, more importantly there is a 12% increase in activating KIR genes and their cognate HLA alleles over control populations (Torres et al., 2012a). These data suggest the interaction of HLA ligand/KIR receptor pairs encoded on two different chromosomes is more significant as a ligand/receptor complex than separately in autism.

## Introduction

Understanding genetic contribution in autism spectrum disorder (ASD) is a major challenge due to genetic complexities of a multifaceted disease. ASD is a puzzling neurodevelopmental condition characterized behaviorally by deficits in social-communication and the presences of restrictive stereotyped behaviors with involvement of genetic, environmental, and neurological components. Although, multiple etiologies are purported to exist, several studies have noted a strong familial clustering suggesting that heredity is of major importance (Bolton et al., 1994; Bailey et al., 1995; Constantino et al., 2013).

The study of human genetics has advanced rapidly over the last 30 years and researchers have adopted new and better methods in an attempt to unravel the extraordinary genomic complexity of autism. An early attempt to find DNA regions that associate with ASD involved the examination of several hundred microsatellites (also known as short tandem repeats) across the genome (Lamb et al., 2000). It became apparent early on that this was not answering questions about autism genetics and this approach was largely abandoned. A significant flaw in these early studies involved the use of siblings as control populations (Philippe et al., 1999). It is now thought that siblings of subjects with autism are unsuitable controls as they have many of the same risk polymorphisms as their autistic siblings. The next attempt involved the studies of purported autism candidate genes. This research typically involved the examination of single nucleotide polymorphisms (SNPs) between subjects and controls in a candidate gene thought to be involved in the etiology of autism. Although a few candidate genes were identified with apparent association to autism, overall the studies hypothesizing the involvement of purported candidate genes were often contradictory and proved to be unreliable (State, 2010).

Genome wide association studies (GWAS) that examine over half a million SNPs have been a little more successful in identifying rare genetic variants (rare variants) in a small number of ASD cases; however, common genetic variants (common variants) have not clearly been identified in GWAS studies (Goldstein, 2009; Manolio et al., 2009; Tao et al., 2016). One interpretation is that rare variants are major contributors to genetic diseases (McClellan and King, 2010) and that ASD involves the interaction of many rare variants, especially in the brain (Irimia et al., 2014; Krystal and State, 2014). The newest research tool involves whole genome sequencing (WGS) to determine the DNA sequence of an individual's entire genome. On the surface this sounds all encompassing; however, weaknesses of this approach besides cost include distorted amplification of certain DNA targets and the difficulty in aligning SNPs to the proper chromosome (Laver et al., 2016).

Several studies have noted a strong familial clustering suggesting that heredity of risk-associated genes is about 50–60% and that about 50% of this risk resides in common variants (Gaugler et al., 2014). The “common variant-common disease” hypothesis was proposed nearly a 100 years ago to explain common diseases with a strong genetic association (Fisher, 1918). This model suggests that each common risk variant (greater or less than 5% of the control population) confers a small degree of risk and that a genetic disease is the result of the combination of several common variants. Common variants (low penetrance) are genetically old and found in multiple populations, whereas rare variants (high penetrance) are new and found in specific populations (Schork et al., 2009). The examination of whole-genome sequence data of autism quartet families (two autism affected siblings and parents) suggested that the majority (69%) of autism affected siblings carried different rare variant mutations (Yuen et al., 2015), supporting the idea that these rare variants are not established in the studied population. Over the years, much attention has been given to discovering the genetic underpinnings of ASD; however, immune abnormalities have consistently been found in this disorder, and recently the number and impact of immune studies has reached a crescendo, including in the area of immunogenetics (Westover et al., 2011; Sweeten and McDougle, 2016; Young et al., 2016).

## Basic immune system

Immunology is the study of a system of molecules, tissue and cells that recognize and attack foreign invaders and abnormal cells that endanger the individual. The entire set of immune genes that contribute to immune function is now referred to as the “Immunome” consisting of about 900 genes across various chromosomes that encode a variety of different proteins to accomplish immune surveillance (Ortutay and Vihinen, 2009).

**Figure 1:**
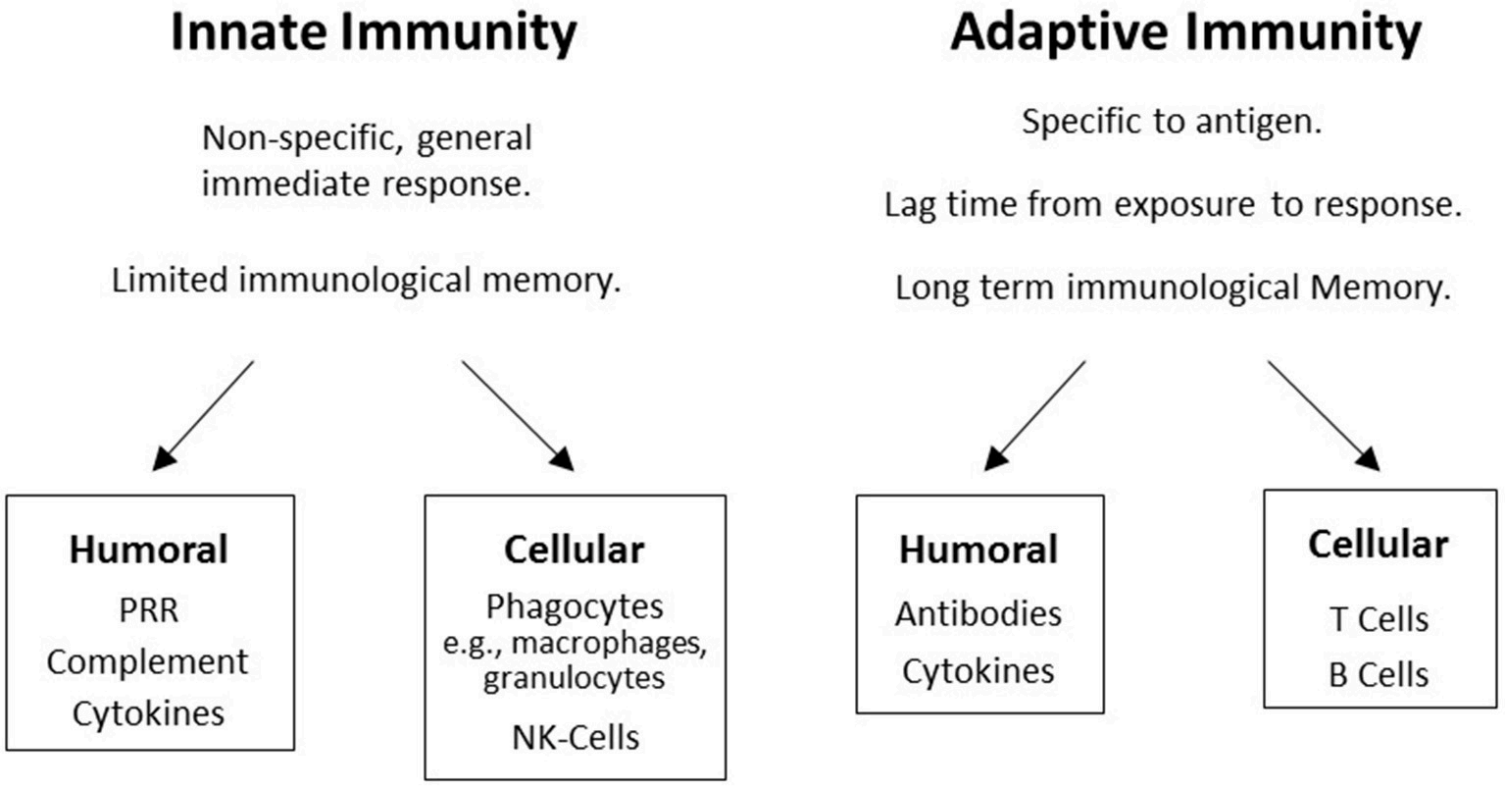
**The two branches of the immune system: Boxes display humoral and cellular components in innate and adaptive immunity**.

A major component of the immune system involves genes in the major histocompatibility complex (MHC) on chromosome 6 in humans. The MHC encodes human leukocyte antigen (HLA) proteins and the terms HLA and MHC are often used interchangeably. The human immune system has two main components, the innate immune system and the adaptive immune system. Both systems have humoral and cellular components (Figure 1), but innate immunity is generally considered to be non-specific whereas humoral immunity provides a specific response to pathogens and foreign antigens. Innate immunity is germ-line encoded, does not adapt after exposure to antigens, and is constitutively expressed leading to an immediate and rapid response to antigens. This is the first line of defense against pathogens, utilizing complement activation and other mechanisms to destroy most microorganisms, typically within hours of exposure.

The classical class I HLA proteins, expressed on essentially all nucleated cells of the body, serve as transducers by presenting antigens to T-cell and B-cell receptors, thereby activating the adaptive immune system for cellular cytotoxicity and antibody production, respectively. The adaptive HLA class II genes produce proteins expressed by specialized immune cells to present antigens to T-cells. HLA-class II genes can recombine by somatic mutation allowing for broad antigen adaptation. HLA-class I and II alleles are some of the most genetically variable coding loci in mammals.

## Innate immunity components

The innate system has soluble complement proteins that help or “complement” the clearance of pathogens in various ways, including acting as an opsonin and by forming a membrane attack complex to kill cells. The complement system contains over 25 proteins and protein fragments that are mainly synthesized in the liver, but also exist in the brain. The innate cellular components include a variety of cell surface and cytoplasmic pattern recognition receptors (PRR) expressed by granulocytes, macrophages, and dendritic cells which bind to pathogen-associated molecular patterns (PAMPs) associated with components of microorganisms. An innate cellular system important in removing aberrant cells (virally infected and transformed) involves the interaction of HLA cognate receptors with killer-cell immunoglobulin-like receptors (KIRs) on NK-cells.

## Adaptive immunity components

HLA proteins are important in presenting antigen to CD4+ (helper T-cells) and CD8+ (cytotoxic T-cells) and the extraordinary diversity in HLA alleles allows for exquisitely sensitive antigen recognition. Adaptive immune responses are slower than those elicited by the innate immune system; however, long term immunological memory is invoked. Memory involves the recognition of antigens from invading organism for extended periods of time, often decades. This is accomplished by the creation of tailor-made memory cells. Antibodies are the main soluble component of the adaptive system; however, cytokines are also important. Memory cells will continue to survive in the body for decades then re-exposure to the antigen activate them to produce the respective antibodies in unlimited numbers. This production of numerous antibodies is accomplished by gene recombination in B-cells followed by a complicated cellular selection process which results in antibodies that are very specific to the foreign antigens. The adaptive immune system is comprised of the cellular components T and B-cells. T-cells have cell-surface receptors that recognize peptides bound to HLA class I and class II proteins, a critical step in cell-mediated immunity.

The cellular components of the adaptive immune system involve T and B-cells. T-cells are important in cell-mediated immunity and are distinguished by the T-cell receptors which recognize short peptides bound to HLA class I and class II proteins. Several subsets of T-cells with distinct functions exist. For example, cytotoxic T-cells seek and destroy virally infected cells and tumor cells while helper T-cells activate macrophages and assist B-cells to mature into antibody producing plasma cells and memory B-cells.

## Immunity and autism

One of the earliest immune associations observed in ASD was by Stubbs et al. (1985) when they noted that pairs of ASD affected children share HLA haplotypes more often than non-affected pairs. The HLA complex is of major interest in medical research as genes/proteins in this region are involved in many immune processes such as organ transplantation, autoimmunity, resistance to specific pathogens, inflammation, ligands for immune cell receptors, and the complement cascade. There is now considerable evidence from association and linkage studies that suggests the involvement of HLA alleles/genes/haplotypes in the etiology of ASD (Torres et al., 2006, 2012a).

## HLA gene complex

**Figure 2:**
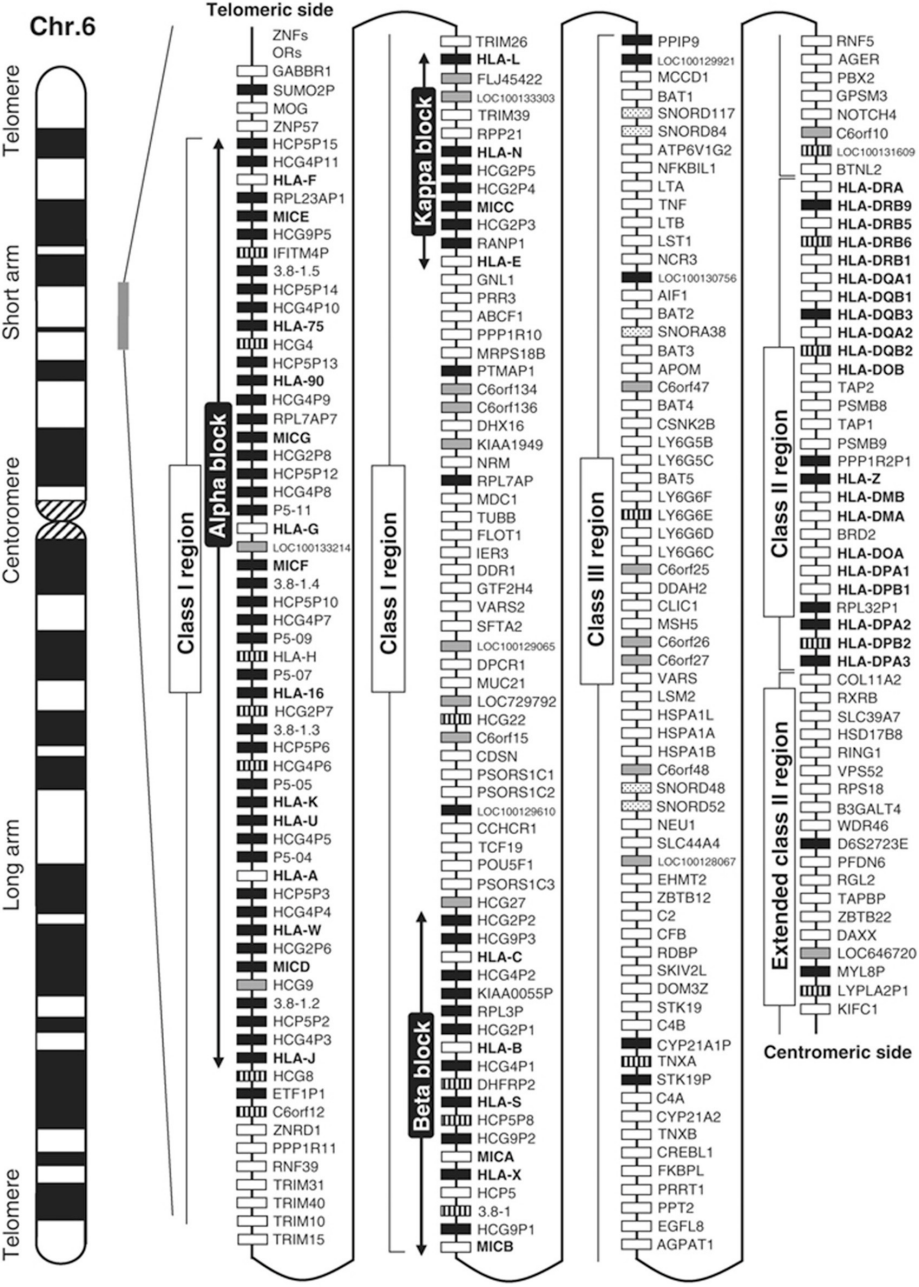
**Gene map of the human leukocyte antigen (HLA) region**. The major histocompatibility complex (MHC) gene map corresponds to the genomic coordinates of 29,677,984 (GABBR1) to 33,485,635 (KIFC1) in the human genome build 36.3 of the National Center for Biotechnology Information (NCBI) map viewer. The regions separated by arrows show the HLA subregions such as extended class I, classical class I, class III, classical class II, and extended class II regions from telomere (left and top side) to centromere (right and bottom side). White, gray, striped and black boxes show expressed genes, gene candidates, non-coding genes and pseudogenes, respectively. The location of the alpha, beta, and kappa blocks containing the cluster of duplicated HLA class I genes in the class I region are indicated. Reprinted with permission from Shiina et al. (2009).

The HLA complex is fascinating as there are about 200 genes that encode proteins with various functions including ligands, receptors, cytokines, signaling factors, heat shock proteins, transcription regulators, and most importantly the recognition of self from non-self. The HLA gene map in Figure 2 (Shiina et al., 2009) illustrates this complexity. The entire 200 HLA gene region is inherited as a single 4.5 million base pair extended haplotype of which there are thousands; most very rare. A genetic haplotype is a contiguous combination of DNA sequences inherited on a single chromosome: eight of the more common extended haplotypes with strong disease associations have been entirely DNA sequenced (Horton et al., 2008). Various extended haplotypes containing millions of base pairs can have identical smaller haplotypes within their borders meaning that they must be carefully examined. Haplotype structure can be important in the understanding of allele-specific events such as protein structure, methylation, outcomes in transplantation, and disease prediction (McQueen et al., 2007). The HLA gene map does not fully illustrate the complexity of the class I and class II regions that have thousands of alleles important in determining self from non-self.

## HLA class I ASD associations

There are numerous ASD associations with alleles/genes in the HLA region. For example, three different research groups have demonstrated a significant association between ASD and the HLA-A2 (A2) allele (Ferrante et al., 2003; Torres et al., 2006; Al-Hakbany et al., 2014). Gourraud et al. (2014) noted that there are over 100,000 SNPs in the 4.5 million base pairs of HLA (1 out of every 45 bases) and concluded that whole genome screening methods like GWAS are not suited for interrogating HLA polymorphisms. They referred to this extreme HLA diversity as a “genome within a genome.” Table 1 illustrates the complexity of SNPs in a small region of the HLA-A-locus. Site specific primers (SSP) PCR assays clearly and inexpensively identify these complex HLA polymorphisms (Bunce and Passey, 2013). Other HLA class I and II loci have similar SNP complexity as do KIR genes, but none of these sets of polymorphisms are readily detected by GWAS techniques.

**Table 1:** **Demonstrates the SNP complexity in HLA alleles**.

**COMPLEXITY OF HLA-A ALLELE SEQUENCES**																						
**Codon position and DNA sequence**																						
	**62**	**63**	**64**	**65**	**66**	**67**	**68**	**69**	**70**	**71**	**72**	**73**	**74**	**75**	**76**	**77**	**78**	**79**	**80**	**81**	**82**	**83**
A^*^01	**CAG**	**GAG**	**ACA**	**CGG**	**AAT**	**ATG**	**AAG**	**GCC**	**CAC**	**TCA**	**CAG**	**ACT**	**GAC**	**CGA**	**GCG**	**AAC**	**CTG**	**GGG**	**ACC**	**CTG**	**CGC**	**GGC**
A^*^02	GG-	- - -	- - -	- - -	- -A	G- -	- - -	- - -	- - -	- - -	- - -	- - -	C- -	- - -	-T-	G- -	- - -	- - -	- - -	- - -	- - -	- - -
A^*^03	- - -	- - -	- - -	- - -	- - -	G- -	- - -	- - -	- -G	- - -	- - -	- - -	- - -	- - -	-T-	G- -	- - -	- - -	- - -	- - -	- - -	- - -
A^*^11	- - -	- - -	- - -	- - -	- - -	G- -	- - -	- - -	- -G	- - -	- - -	- - -	- - -	- - -	-T-	G- -	- - -	- - -	- - -	- - -	- - -	- - -
A^*^23	G- -	- - -	- - -	G- -	- -A	G- -	- - -	- - -	- - -	- - -	- - -	- - -	- - -	- - -	-A-	- - -	- - -	C- -	-T-	GC-	-T-	C- -
A^*^24	G- -	- - -	- - -	G- -	- -A	G- -	- - -	- - -	- - -	- - -	- - -	- - -	- - -	- - -	-A-	- - -	- - -	C- -	-T-	GC-	-T-	C- -
A^*^25	-G-	A-C	- - -	- - -	- - -	G- -	- - -	- - -	- - -	- - -	- - -	- - -	- - -	- - -	-A-	-G-	- - -	C- -	-T-	GC-	-T-	C- -
A^*^26	-G-	A-C	- - -	- - -	- - -	G- -	- - -	- - -	- - -	- - -	- - -	- - -	- - -	- - -	- - -	- - -	- - -	- - -	- - -	- - -	- - -	- - -
A^*^29	-T-	C- -	- - -	- - -	- - -	G- -	- - -	- - -	- -G	- - -	- - -	- - -	- - -	- - -	- - -	- - -	- - -	- - -	- - -	- - -	- - -	- - -
A^*^30	- - -	- - -	- - -	- - -	- - -	G- -	- - -	- - -	- -G	- - -	- - -	- - -	- - -	- - -	-T-	G- -	- - -	- - -	- - -	- - -	- - -	- - -
A^*^31	- - -	- - -	- - -	- - -	- - -	G- -	- - -	- - -	- - -	- - -	- - -	-T-	- - -	- - -	-T-	G- -	- - -	- - -	- - -	- - -	- - -	- - -
A^*^32	- - -	- - -	- - -	- - -	- - -	G- -	- - -	- - -	- - -	- - -	- - -	- - -	- - -	- - -	-A-	-G-	- - -	C- -	-T-	GC-	-T-	C- -
A^*^33	-G-	A-C	- - -	- - -	- - -	G- -	- - -	- - -	- - -	- - -	- - -	-T-	- - -	- - -	-T-	G- -	- - -	- - -	- - -	- - -	- - -	- - -
A^*^34	-G-	A-C	- - -	- - -	- -A	G- -	- - -	- - -	- -G	- - -	- - -	- - -	- - -	- - -	-T-	G- -	- - -	- - -	- - -	- - -	- - -	- - -
A^*^36	- - -	- - -	- - -	- - -	- - -	- - -	- - -	- - -	- - -	- - -	- - -	- - -	- - -	- - -	- - -	- - -	- - -	- - -	- - -	- - -	- - -	- - -
A^*^43	-T-	C- -	- - -	- - -	- - -	G- -	- - -	- - -	- - -	- - -	- - -	- - -	- - -	- - -	- - -	- - -	- - -	- - -	- - -	- - -	- - -	- - -
A^*^66	-G-	A-C	- - -	- - -	- - -	G- -	- - -	- - -	- -G	- - -	- - -	- - -	- - -	- - -	-T-	G- -	- - -	- - -	- - -	- - -	- - -	- - -
A^*^68	-G-	A-C	- - -	- - -	- - -	G- -	- - -	- - -	- -G	- - -	- - -	- - -	- - -	- - -	-T-	G- -	- - -	- - -	- - -	- - -	- - -	- - -
A^*^69	-G-	A-C	- - -	- - -	- - -	G- -	- - -	- - -	- -G	- - -	- - -	- - -	- - -	- - -	-T-	G- -	- - -	- - -	- - -	- - -	- - -	- - -
A^*^74	- - -	- - -	- - -	- - -	- - -	G- -	- - -	- - -	- - -	- - -	- - -	- - -	- - -	- - -	-T-	G- -	- - -	- - -	- - -	- - -	- - -	- - -
A^*^80	G- -	- - -	- - -	- - -	- - -	G- -	- - -	- - -	- - -	- - -	- - -	- - -	A- -	- - -	- - -	- - -	- - -	- - -	- - -	- - -	- - -	- - -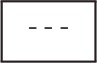 = same as reference sequence (A^*^01)												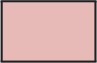 = different from reference sequence (A^*^01)										

*Approximately one out of three DNA bases in this sequence can be a polymorphism*.

It has recently been reported in the Thai population that certain HLA-B alleles (B13:02, B35:02, B44:03, and B56:01) are significantly associated with ASD while two others (B18:02 and B46:12) are associated in a protective manner (Puangpetch et al., 2015). This is not surprising as certain HLA extended haplotypes which have about 200 genes including specific HLA-A, B, and C alleles have very strong associations with ASD. For example, in the Caucasian population HLA-B44 and B15 alleles as part of extended haplotypes 44.1 and 62.1, respectively, are associated with ASD (Torres et al., 2012a).

HLA-G non-classical I molecules are expressed during pregnancy on trophoblast cells that interact with leukocyte-associated immunoglobulin-like receptor (LAIR) and KIR (2DL4) molecules expressed by maternal NK-cells at the uterine fetal/maternal interface to suppress normal immune responses (Tilburgs et al., 2015). A 14 bp deletion in the HLA-G 3′-UTR is associated with higher levels of HLA-G expression whereas a 14 bp insertion associates with reduced HLA-G levels. Guerini et al. (2015) have shown that ASD subjects have an increase in the 14 bp insertion and lower levels of soluble HLA-G protein. This suggests that ASD subjects and their mothers have less HLA-G-mediated immune tolerance during pregnancy. Human non-classical HLA I molecules could be important in brain development as it has recently been shown that non-classical MHC class I molecules are expressed in the olfactory bulb, hippocampus, cerebellum, and nerve nuclei in the developing embryonic mouse brain (Liu et al., 2015).

**Figure 3:**
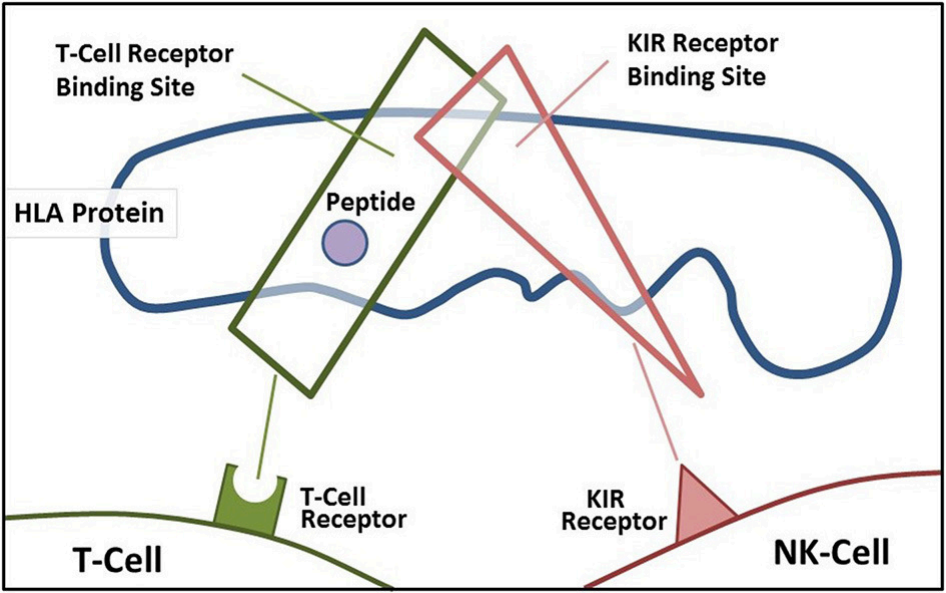
**Demonstrates the overlapping regions for KIR ligand binding and antigen-presenting portions of the HLA protein**.

**Figure 4:**
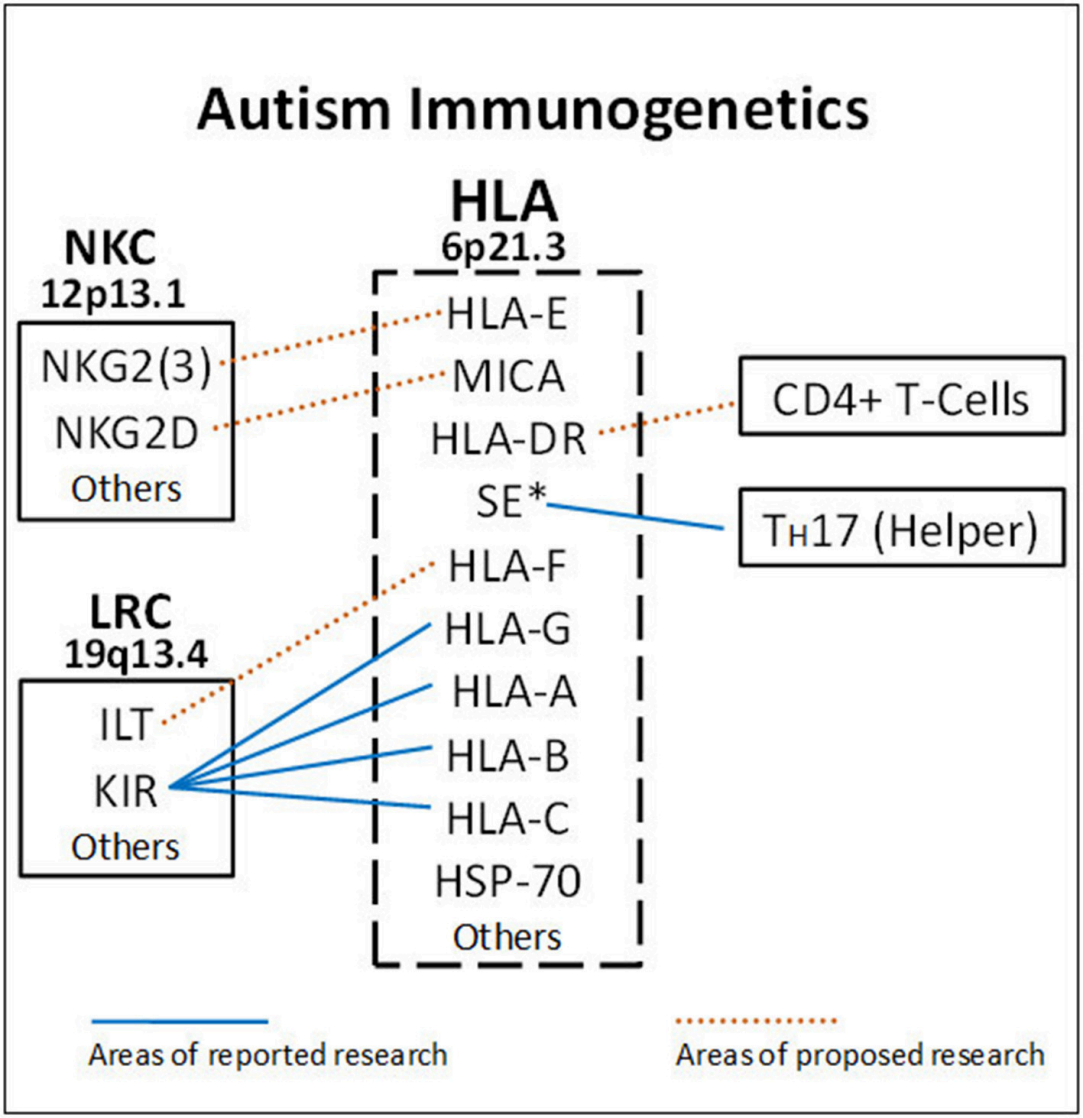
**HLA gene complex encode ligands that react with receptors on the Leukocyte Receptor Complex (LCR) and Natural Killer Cell Gene Complex (NKC) to influence killing**. Blue lines indicate areas of completed research. HLA-A, B, C ligands bind to various KIR receptors encoded on chromosome 19. The DRB1^*^0401 shared epitope (SE^*^) is strongly associated with ASD (RR = 19.8) and helps increase TH17 cells. The dashed lines suggest future ASD research to get a more complete picture on NK-cell killing and antibody production. HLA-DR alleles bind to CD4+ helper T-cells to influence B-cells. HLA-E and MHC Class I like- sequence A (MICA) proteins are ligands for activating molecules NKG2(3) and NKG2D, respectively, that influence NK-cell killing (chromosome 12).

A main function of HLA class I molecules is to present antigens to CD8+ T-cells, thus starting the complex immune process that creates cytotoxic T-cells to attack specific targets. Any gene in an extended haplotype may be the candidate ASD gene and other HLA alleles may be passengers, however, the identification of foreign peptides that bind to A2 could suggest microorganisms that may be involved in the etiology of ASD. Our data suggests that the HLA class I proteins cognate ligands for KIR are important in autism and that other NK-cell killing receptors could also be important (Figure 4). This interaction does not involve peptide binding as KIR receptors recognize specific amino acid sequences of certain HLA cognate protein ligands (Parham and Moffett, 2013). HLA class I A, B, C alleles all behave as antigen presenting ligands for self and nonself-peptides; however, only certain HLA-A, B, C alleles are ligands for KIR cell surface proteins. Although the HLA cognate ligand site slightly overlaps the peptide binding site, it has a separate function (Figure 3). Specific HLA ligands that bind to KIR activating receptors have been shown to be increased in ASD (Torres et al., 2012b, 2016; Guerini et al., 2014). It should be mentioned that the HLA-A2 does not behave as a KIR ligand.

It was only a few years ago that the brain was considered immune privileged, being protected from immune fluctuations by the blood brain barrier. Today there is increasing evidence that the systemic immune system is involved in CNS functioning. Pro-inflammatory cytokines such as TNF-α can easily cross the blood brain barrier and complement proteins such as C1q and C4 are active in brain function (Johnson et al., 1994; Torres et al., 2001). C1q appears to be especially important in eliminating unwanted synapses (Stevens et al., 2007). Perhaps the most important research from an immune-brain perspective is the involvement of HLA class I proteins in brain development. The expression of HLA class I proteins occurs throughout the brain on neurons and even synaptic membranes (Elmer et al., 2013). It is yet unknown if there are differences in class I alleles in brain development and this is an area clearly deserving of further research.

## HLA class II ASD associations

A maternal-fetal immune interaction has been suggested by Lee et al. (2006) in which boys with autism and their mothers were shown to have an increased DR4 frequency over the control subjects (odds ratios 4.20 and 5.54, respectively). Johnson et al. (2009) later reported significant transmission disequilibrium for HLA-DR4 (odds ratio 4.67) from maternal grandparents to mothers of children with autism, more evidence that HLA-DR4 has a maternal-fetal interaction. Finally, it has been shown in Han Chinese that the HLA-DRB1 allelic frequencies including DR4 are different in autism subjects vs. control subjects (Chien et al., 2011). It should be noted that it is common for racial groups to have different HLA alleles associated with autoimmune diseases.

Warren et al. (1996) also noted that the third hypervariable region (HVR-3) of certain class II DR4 alleles, referred to as the shared epitope (SE), were strongly associated with ASD (*p* < 0.01; relative risk = 19.8). In this review, odds ratio and relative risk, two similar mathematical models to examine disease risk, are considered equivalent. The HLA-DRB1 SE is a 5 amino acid motif shared by 5 DRB1 alleles. The SE also strongly associates with the majority of severe rheumatoid arthritis (RA) patients. The basis for the SE association with ASD and RA is unknown; however, it has been proposed that the SE peptide acts as a signal transduction ligand that activates nitric oxide (NO) and reactive oxygen species production (de Almeida et al., 2011). It is important to mention that De Almeida et al. (2010) concluded, in an earlier publication, that the HLA DRB1 shared epitope peptide is a potent immune-stimulatory ligand that polarizes naive helper T-cell toward the potent inflammatory T_H_17 lineage resulting in higher IL-17 levels detected in autism subjects (Al-Ayadhi and Mostafa, 2012; Onore et al., 2012).

It should be noted that mothers with SE alleles on either chromosome were more likely to give birth to an ASD child, referred to by Warren et al. (1996) as a “maternal attack against fetal tissue.” The SE observation is important as it suggested that small peptide epitopes contributed to the high relative risk in ASD years before the T_H_17 cell observation was made.

## Autoantibodies in autism

One of the most interesting areas of current ASD research is the observation in at least 8 studies that up to about 10% of mothers with ASD children and only 0–2% of controls have humoral antibodies against fetal brain proteins (Croen et al., 2008; Braunschweig and Van de Water, 2012). Because HLA class II molecules are important in antibody production it would be interesting to know if there is an association between mothers who make these antibodies and certain DRB1 alleles. The entire area of maternal antibodies against fetal proteins, the production of autoantibodies, as well as antibodies to microbial pathogens should be studied more in ASD (Grether et al., 2016).

## Class III ASD associations

Four of the 25 proteins in the complement system that help or “complement” antibodies and phagocytic cells to clear pathogens from the organism are encoded within the HLA complex (C4A, C4B, C2, and Bf). The C4 complement proteins (C4A and C4B) are encoded by two separate genes in the HLA class III region (Torres et al., 2001). It was reported 25 years ago that subjects with autism had a significant increase in the deletion of the HLA class III C4B gene (C4B null allele) compared to control subjects (Warren et al., 1991; Odell et al., 2005). ASD subjects were also noted to have a significant deficiency in the plasma C4B protein as determined by an Elisa assay (Warren et al., 1994). Mostafa and Shehab (2010) have also reported a significant increase in the deletion of the C4B gene in the Egyptian ASD population. However, the gene copy number data from qPCR assays do not entirely agree with the results from the C4B protein assay, a not uncommon occurrence when comparing genomic and protein expression data (Kendrick, 2014), thus the solidity of these results are still uncertain. While the data of Warren et al. (1996) suggest that the C4B null allele is another common variant, as it is part of the 44.1 extended haplotype that also contains the SE, further clarification of the association of C4B null alleles is required, preferably using samples with known C4A/C4B DNA complotypes.

There are four proinflammatory cytokines: tumor necrosis factor-alpha (TNFα), lymphotoxin alpha and beta (LTA and LTB), and leukocyte specific transcript-1 (LST1) in the class III region that are important in inflammation and infection. TNFα is a proinflammatory, multifunctional cytokine that is synthesized in numerous cells including NK-cells, macrophages, granulocytes and T-cells. In the CNS, TNFα is made in NK-cells, microglia (brain macrophages), astrocytes, and neurons and is necessary for neural cell differentiation and neuron maturation and may be critical for proper synaptic pruning (Cacci et al., 2005). Elevated levels of TNFα are present in ASD patients, as well as in numerous neurological disorders including multiple sclerosis, Alzheimer's Disease, Parkinson's Disease, ischemia, and traumatic brain injury (Montgomery and Bowers, 2012). The three adjacent proinflammatory cytokine genes: lymphotoxin alpha and beta (LTA and LTB) and leukocyte specific transcript-1 (LST1) have not been studied as extensively as TNFα (Ovsyannikova et al., 2010) and deserve further investigation.

There is a developing consensus that there are elevated plasma levels of proinflammatory cytokines such as IL-1β, IL-6, IL-8, as well as CCL2 and CCL5 chemokines in ASD (Grigorenko et al., 2008; Ashwood et al., 2011). Decreased levels of anti-inflammatory cytokines, most notably TGF-β and IL-10, have also been reported in ASD (Okada et al., 2007; Abdallah et al., 2013; Jyonouchi et al., 2014). In addition to these proinflammatory cytokines, it is interesting to note that an increase in interferon-gamma (INFγ) and TNFα has been observed in brain tissue and cerebral spinal fluid of in ASD subjects (Li et al., 2009). These two cytokines are of special interest, as TNFα is in the HLA class III region and activates NK-cells to make large amounts of INFγ (Schoenborn and Wilson, 2007). TNFα also may be important in ASD as it appears to be a key mediator in neuroinflammation and blood-brain barrier deterioration (McCoy and Tansey, 2008). TNFα concentrations are also elevated in several brain disorders including multiple sclerosis, Alzheimer's Disease, Parkinson's Disease, ischemia, and traumatic brain injury (Montgomery and Bowers, 2012). It is unclear if TNFα contributes to the disease state or if the higher concentrations limit brain damage. Finally, another avenue for investigation, is the class III gene encoding the heat shock protein 70 (HSP70) which is induced in many CNS disorders such as stroke, epilepsy, trauma and ASD (El-Ansary and Al-Ayadhi, 2012).

INFγ may contribute to ASD pathology by increasing the enzyme nitric oxide synthase in astrocytes and microglia, and thereby increasing brain levels of NO. It is thought that activated immune cells secrete high levels of the free radical NO to damage pathogens. In the brain however, NO is typically produced by neurons and it acts as an intercellular messenger modulating synaptogenesis, dendrite, and axonal growth and neuronal release of various neurotransmitters (Hess et al., 1993; Lizasoain et al., 1996). Problems with synaptic development and plasticity are implicated in the neuropathology of ASD (Ebert and Greenberg, 2013).

It was reported a couple of years ago that serum levels of IL-17 were elevated in ASD subjects over control subjects suggesting that T helper 17 (T_H_17) cells are involved in the etiology of ASD (Al-Ayadhi and Mostafa, 2012; Onore et al., 2012). T_H_17 cells are CD4+ helper T-cells characterized by a unique cytokine profile, mainly high levels of IL-17 thought to be critical for the development of autoimmunity (Bedoya et al., 2013).

## The importance of HLA immunity in autism

The HLA gene complex has been considered by many to be the most important region for immune function in the human genome. However, the system is not perfect and certain HLA allotypes and haplotypes have been shown to be strongly associated with a wide spectrum of autoimmune diseases, such as Type I diabetes, celiac disease, psoriasis, rheumatoid arthritis (RA), lupus erthymatosis, myasthenia gravid, Sjogren Syndrome, and now ASD. The HVR-3 (or shared epitope) that is increased in ASD (Warren et al., 1996) is also associated with several autoimmune diseases such as RA, psoriatic arthritis, and systemic lupus erythematosus (de Almeida et al., 2011). Various HLA disease associations often have the relative risk factors 15 to 20-fold higher in subjects with autoimmune diseases than control subjects (Feitsma et al., 2008). The HVR-3 (DRB1^*^0401) association with ASD had a comparable relative risk of 19.8 and 31.5% increase in the ASD population relative to control subjects, fitting the criteria for a “common variant–common disease” association.

## HLA/KIR common genetic variants

There is currently an intense interest in identifying genetic variants that increase the risk of developing ASD. The goal of this paper is to summarize data which suggests that certain HLA/KIR immune alleles/genes fit the criteria as common genetic variants. For example, the HLA-A2 allele in independent studies from three different groups has a >10% increased frequency in the ASD population than controls (Table 2A). Interestingly, it was over three decades ago that Stubbs and Magenis (1980) first implicated HLA-A2 in ASD. A 14 base pair insertion in the class I non-classical HLA-G loci is strongly associated with ASD (Guerini et al., 2015) and is clearly a common variant at 18% over controls (Table 2A).

**Table 2:** **(A) Suggests that HLA class I and class II alleles are common genetic variants (>5%). (B) Suggests that KIR genes and KIR gene-content haplotypes are common genetic variants**.

**Variables**	***p*-value**	**Odds ratio**	**Subjects (%)**	**Controls (%)**	**Common variant (>5%)**
**(A) HLA**					
**Class I alleles**					
A2^1^	0.0002	2.22	101/258 (39.1%)	75/265 (28.3%)	10.8
A2^2^	0.001	3.02	30/70 (42.8%)	54/200 (27.0%)	15.8
G 14 bp(+/+)^3^	0.01	2.70	19/74 (25.7%)	3/39 (7.7%)	18.0
**Class II alleles**					
DR4^4^	0.003	3.24	10/32 (31.3%)	117/950 (12.3%)	19.0
DR4^5^	0.004	1.60	50/205 (24.4%)	68089/406503(16.8%)	7.6
SE^6^	<0.001	19.80	17/50 (34.0%)	2/79 (2.5%)	31.5
**Class III alleles**					
C4BQ0^6^	0.03	4.60	27/50 (54.0%)	16/79 (20.2%)	13.8
**(B) KIR**					
**Kir genes**					
3DS1^7^	0.004	1.89	83/158 (52.5%)	65/176 (36.9%)	15.6
2DS1^7^	0.00007	2.45	95/158 (60.1%)	67/176 (38.1%)	22.0
2DS2^8^	0.030	1.83	63/90 (70.0%)	140/250 (56.0%)	14.0
aKIR^*^^7^	0.0004	1.36	126/158 (79.7%)	128/176 (72.7%)	7.0
**Kir haplotypes**					
cA01^9^	0.00001	0.53	110/178 (61.8%)	6248/9024 (69.2%)	−7.4 (protective)
cB01^9^	0.005	1.69	41/178 (23.0%)	1184/9024 (13.1%)	9.9
cB01/tA01^9^	0.002	2.06	25/170 (14.7%)	697/9024 (7.7%)	7.0
**Kir genes** + **HLA ligands**					
aKIR + HLA^7^	0.0006	1.56	117/155 (75.5%)	104/164 (63.4%)	12.1

1 *Torres et al., 2006*.

2 *Al-Hakbany et al., 2014*.

3 *Guerini et al., 2015*.

4 *Lee et al., 2006*.

5 *Torres et al., 2002*.

6 *Warren et al., 1996*.

7 *Torres et al., 2012b*.

8 *Guerini et al., 2014*.

9 *Torres et al., 2016*.

The DR4 allele has higher frequencies of 18.94 and 7.64% in two ASD populations over control populations suggesting that DR4 is a common variant (Table 2A). Warren et al. (1996) presented data that strongly suggested that the shared epitope is a common genetic variant with a 31.5% increase of ASD over control subjects (Table 2A).

**Figure 5:**
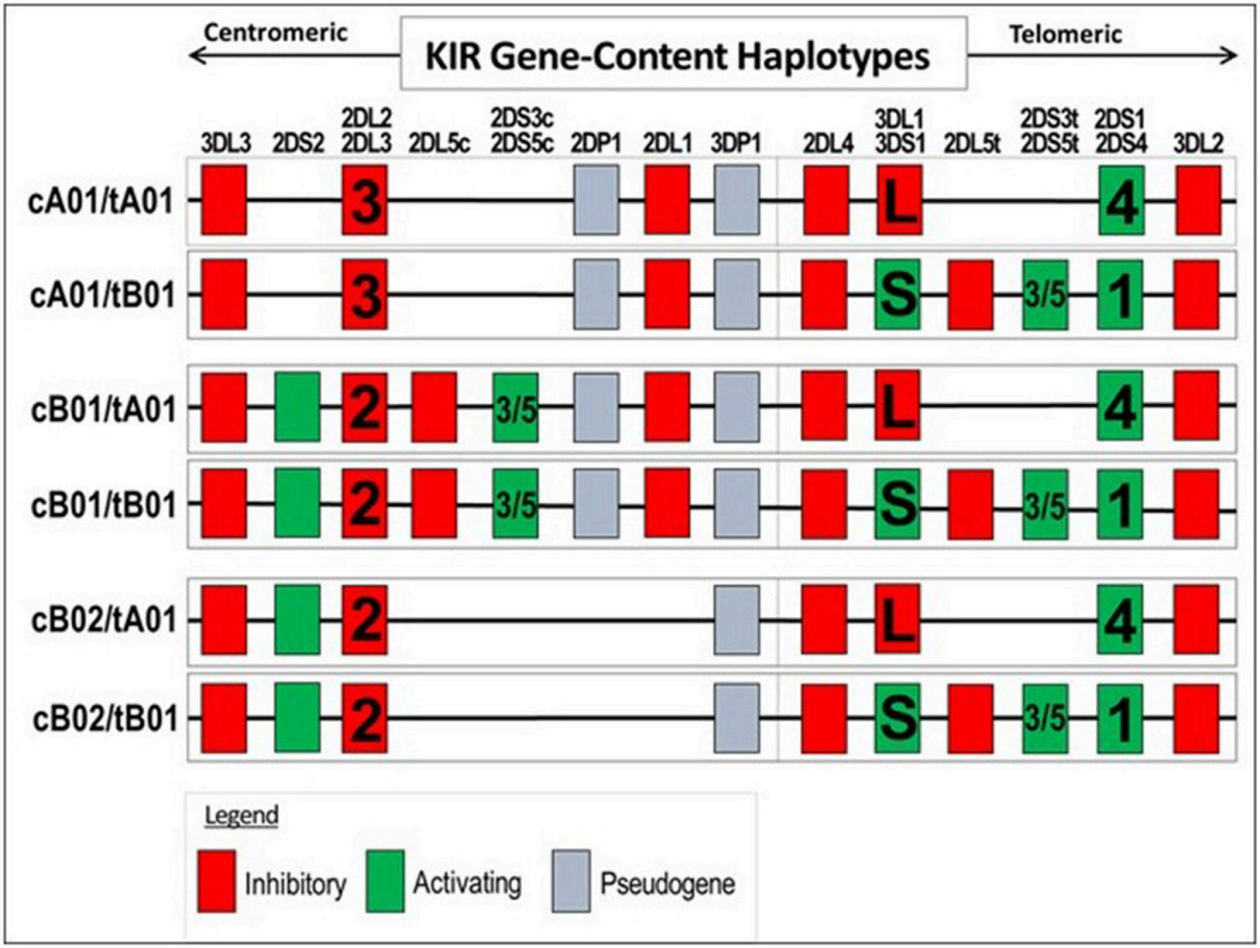
**Genes present in KIR gene-content haplotypes**. Only genes present in a haplotype are listed under the gene column: for example, 2DS2 is absent in the first two haplotypes, but present in the last four haplotypes. Certain haplotype can have 2DL2 or 2DL3 and other haplotype can have 2DL1 or 2DS1 and 2DS1 or 2DS4. The complete gene-content KIR haplotype (cB01/tA01) is a proposed common variant.

Examination of KIR gene frequencies suggests that certain activating genes are common variants with ASD frequency differentials well above 5% in two different publications (Torres et al., 2012b; Guerini et al., 2014). The KIR region in the leukocyte receptor complex (LRC) is not typical as KIR haplotypes are based on different combination of activating and inhibiting genes that influence NK-cell killing (Pyo et al., 2013; Torres et al., 2016). Gene frequency analyses suggest that three activating genes (3DS1, 2DS1, and 2DS2) are common variants (Table 2B). Additionally, examinations of all activating KIR genes (aKIR) indicate an increase in total activating genes in ASD over control subjects. KIR gene-content haplotypes have centromeric and telomeric parts that join at the junction between 3DP1 and 2DL4 (Figure 5). Examination of the KIR haplotypes suggests that three partial KIR haplotypes (tA01/cA01/cB01) and a complete KIR gene-content haplotype (cB01/tA01) all meet common variant criteria (Table 2B; Torres et al., 2016).

## Autism immunogenetics

Immunogenetics commonly refers to the strong association of HLA alleles/genes with autoimmune diseases (Shiina et al., 2004). We wish to propose that much of the immune dysfunction in ASD has a basis in the HLA /KIR gene complexes; thus, we have coined the term Autism Immunogenetics. Although, many of the multiple thousands of HLA alleles are rare there are hundreds of weIl-characterized HLA class I and class II alleles that make up a significant part of the genetic diversity of the 900 gene Immunome.

One must remember that the entire 200 genes in the HLA gene complex are inherited as large 4.5 million base pair extended haplotypes and a couple of these large extended haplotypes have been associated with autism (Warren et al., 1996). The association of the A2, DR4 alleles and C4B null allele with ASD may be due to the fact that they are part of the extended haplotype 44.1 that is ASD associated (Figure 4; Table 2). However, the important observation is that all are common genetic variants. It would be interesting to know which extended haplotypes have the HLA-G 14 bp insertion as it strongly associates with ASD. The shared epitope peptide may be the most important as it directs helper T-cells to the T_H_17 lineage which appears to be important in the pathogenesis of autoimmune neuroinflammatory diseases including ASD (Figure 4; Onore et al., 2012; Choi et al., 2016). A recent publication suggested that maternal T_H_17 cells could induce an ASD-like phenotype in newborn mice and that blocking antibodies to IL-17a ameliorated these symptoms (Choi et al., 2016). The high relative risk for the SE in ASD noted by Warren et al. (1996) may be due to an increase in T_H_17cells.

## Concluding remarks

A significant body of evidence has accumulated over the past 30 plus years to demonstrate that specific variants in immune system genes, involving both innate and adaptive immunity pathways, are associated with the incidence of ASD. However, such associations have not been detected by genome wide screening methods, e.g., GWAS, and therefore have been widely ignored by the majority of investigators in the autism research field. The inability to detect immune gene ASD associations by GWAS techniques is fully understandable, considering the massive genetic complexity of the “Immunome,” with more than one hundred thousand polymorphisms localized within a small segment (<5%) of the total genome. Thus, highly focused methods, usually PCR or sequence-based typing, are required to reveal disease associations with immune genes or haplotypes, and these can usually be detected using relatively small number of patient and control samples. Next-Generation Sequencing methods to rapidly sequence the HLA locus are well developed for such autism immunogenetic studies (Nelson et al., 2015; Carapito et al., in press). Indeed, as summarized in Table 2, several HLA and KIR gene variants that meet the “common variant–common disease” criteria have been identified using relatively small sample sizes: in the low hundreds. Perhaps the most interesting observation concerning common genetic variants is the 12% increase of the HLA ligand /KIR activating gene complex in autism over the control populations (Table 2). We argue strongly that additional efforts be focused on identifying and characterizing the role of immune genes in the etiology of ASD, particularly using phenotypically well-defined populations of autistic subjects.

The data summarized in this review raises numerous questions in addition to those raised earlier in this paper. For example, how do different immune gene variants influence brain development and function? While it is clear that many HLA proteins are expressed in or on neural cells and they can modulate the activity of downstream effector cells or proteins, such as NK-cells or pro-inflammatory cytokines, details on how they actually function are unknown. The fact that a single amino acid change in certain HLA class I and II proteins can alter their functionality makes such studies extremely challenging. For example, single DNA base differences in HLA-C alleles encode different amino acids which are ligands for different KIR receptors (below) (Torres et al., 2016).

**Table:** 

DNA	Ligand	Receptor
Codon AAC	HLA-C amino acid #80 ASN —————-	KIR-2DS2 and 2DL2
Codon AAA	HLA-C amino acid #80 LYS —————-	KIR-2DS1 and 2DL1

An additional question that needs to be addressed is why do up to 10% of mothers with ASD children have humoral antibodies against fetal brain proteins (compared to 0–2% of control mothers) and do these autoantibodies adversely affect fetal brain development? Furthermore, does the 14 bp insertion in the HLA G gene that occurs more frequently in ASD subjects and their mothers impair immune tolerance during pregnancy and increase the risk of *in utero* developmental problems?

## HLA alleles and KIR genes as common genetic variants in ASD

The observations that several HLA alleles as well as several KIR genes and haplotypes appear to be common genetic variants associated with ASD (Table 2) raises the question as to whether or not other immune genes (for example HLA-E, HLA-F, and MICA) have similar ASD associations. These issues should be addressed in future studies. In summary, by further understanding how immune gene variants participate in the etiology of ASD, it may be possible to: #1 develop biological markers to predict ASD at an earlier stage or even *in utero* and #2 develop targeted pharmaceutical molecules such as monoclonal antibodies, decoy peptides, and special nucleic acid molecules against SE, HLA, and KIR molecules.

## Author contributions

ART conceived of the study, participated in its design and coordination, and drafted the manuscript. TS and JW participated in data collection and helped draft the manuscript. PB, DW, CD, AJT, DO, and LC helped draft the manuscript. RJ performed the statistical analyses. MB participated in data collection and statistical analyses. All authors read and approved the final manuscript.

### Conflict of interest statement

The authors declare that the research was conducted in the absence of any commercial or financial relationships that could be construed as a potential conflict of interest.
